# Calendar

**DOI:** 10.1155/S1463924685000232

**Published:** 1985


Journal of Automatic Chemistry/*Journal of Clinical Laboratory Automation*, Vol. 7, No. 2 (Apr–Jun 1985), pp. 102–103

### Calendar

### MAY 1985

### 6th ECCLS Seminar

To be held from 8 to 10 May in Cologne, FR Germany.

*Further information from ECCLS Central Office, Wellcome Research Laboratories, Beckenham, Kent BR3 3BS, UK.*

### Moisture Measurement in Solids and Gases

Course to be held from 14 to 15 May in Chislehurst, UK.

*Further information from Sira Ltd, South Hill, Chislehurst, Kent BR7 5EH, UK.*

### JUNE 1985

### Hewlett-Packard 1985 Analytical Symposium

To be held from 10 to 14 June 1985 at the Moat House, Stratford-upon-Avon, UK.

*Further information from Mrs Tina Mears, Hewlett-Packard Ltd, Miller House, The Ring, Bracknell, Berkshire, UK.*

### Budapest Chromatography Symposium: the 5th American–Eastern European Symposium on Liquid Chromatography

To be held from 11 to 14 June in Budapest, Hungary.

*Further information from Congress Bureau Motesz, Budapest, PO Box 32, H-1361, Hungary.*

### Product Design Assurance in Engineering

To be held from 11 to 13 June 1985 at the Wembley Conference Centre, London.

*Further information from Mrs H. M. W. Gibbons, Society of Environmental Engineers, Owles Hall, Buntingford, Hertfordshire, UK.*

### Computing in Clinical Laboratories

To be held from 12 to 14 June in Stuttgart, FR Germany.

*Further information from The Chairman of the Fifth Conference CCL '85, PD Dr Chr Trendelenburg, Katharinenhospital, Klinisch-Chemisches Institut, Kriegsbergstraße 60, D-7000 Stuttgart 1, FR Germany.*

### Developments in Analytical Methods in the Pharmaceutical and Biomedical Sciences

Organized by the Italian Group for Mass Spectrometry in Biochemistry and Medicine and by the Department of Chemical Sciences of the University of Camerino. To be held in Camerino, Italy from 17 to 20 June.

*Further information from Dr Alberto Frigerio, Italian Group for Mass Spectrometry in Biochemistry and Medicine, Via Eustachi, 36 20129, Milan, Italy.*

### Canadian Congress on Laboratory Medicine

To be held from 22 to 26 June at the Westin Hotel, Edmonton, Alberta, Canada.

*Further information from Dr Donald G. Young, CCLM 85, Misericordia Hospital, 16940-87 Avenue, Edmonton, Alberta T5R 4HS, Canada.*

### JULY 1985

### Workshop on Liquid Scintillation Counting

To be held from 15 to 19 July in Loughborough, UK.

*Further information from Dr P. Warwick, Department of Chemistry, Loughborough University of Technology, Loughborough, Leicestershire LE11 3TU, UK.*

### 37th Annual Meeting of the American Association of Clinical Chemistry

To be held from 21 to 26 July in Atlanta, USA.

*Further information from Meetings Department AACC, 1725 K. St. N.W., Suite 1010, Washington, D.C. 20006.*

### Infra-red Summer School

To be held from 29 July to 2 August in Cambridge, UK.

*Further information from Mrs R. Fullerton, Pye Unicam Ltd, York Street, Cambridge CB1 2PX, UK.*

### AUGUST 1985

### 5th International Meeting on Clinical Laboratory Organization and Management

To be held from 27–29 August in Haifa, Israel.

*Further information from Dr E. Shafkir (over).*

### SEPTEMBER 1985

### Sixth European Congress of Clinical Chemistry

To be held from 1–5 September in Jerusalem, Israel.

*Further information from Dr E. Shafkir, Chairman of the Co-ordinating Board: Clinical Chemistry and Enzymology Meetings, P.O. Box 50006, Tel-Aviv, Israel.*

### 2nd Congress of the International Society of Animal Clinical Biochemistry

To be held from 2–6 September in Jerusalem, Israel.

*Further information from Dr E. Shafkir (above).*

---

### 5th International Congress on Clinical Enzymology

To be held from 3–6 September in Jerusalem, Israel.

*Further information from Dr E. Shafkir (above).*

---

### Flow Analysis III

To be held from 5 to 8 September in Birmingham, UK.

*Further information from Dr A. M. G. Macdonald, Department of Chemistry, The University, PO Box 363, Birmingham B15 2TT, UK.*

---

### 14th X-Ray Spectrometry Conference

To be held from 9 to 13 September at Durham University.

*Further information from Mrs Margaret Courtney, Pye Unicam Ltd, York Street, Cambridge CB1 2PX, UK.*

---

### IUPAC 1985

To be held from 9 to 13 September 1985 in Manchester, UK.

*Further information from the Royal Society of Chemistry, Burlington House, London W1V 0BN.*

### Colloquium Spectroscopicum Internationale XXIV

To be held from 15 to 21 September 1985 in Garmisch-Partenkirchen, FR Germany.

*Further information from CSI XXIV, Organisationsbüro, Institut für Spektrochemie und angewandte Spektroskopie, Postfach 778, D4600 Dortmund 1, FR Germany.*

---

### 12th Annual Meeting of the Federation of Analytical Chemistry and Spectroscopy Societies

To be held from 29 September to 4 October in Philadelphia, Pennsylvania, USA.

*Further information from Allan Ullman, Procter & Gamble Company, 6250 Center Hill Road, Cincinnati, Ohio 45224, USA.*

---

### Sixth Colloquium on Prospective Biology

To be held from 30 September to 4 October at Pont-à-Mousson, France.

*Further information from Association Loi 1901 Siege Social, 7 rue Albert-Lebrun, F 54000 Nancy, France.*

---

### OCTOBER 1985

### 35th Canadian Chemical Engineering Conference

To be held from 6 to 9 October at the Calgary Convention Centre, Canada.

*Further information from Dr Roger M. Butler, Department of Chemical and Petroleum Engineering, University of Calgary, Calgary, Alberta T2N 1N4, Canada.*

### DECEMBER 1985

### Chem Asia 85

To be held from 2–5 December in Singapore.

*Further information from Chatsworth House, 59 London Road, Twickenham TW1 3SZ, UK.*

---

### 1986 MEETINGS

### Fourth World Filtration Congress

To be held from 22 to 25 April 1986 in Ostend, Belgium.

*Further information from the Secretariat of World Filtration Congress, IV, KVIV-Technologisch Instituut, Jan van Rijswijcklaan 58, B 2018 Antwerpen, Belgium.*

---

### Analytica 86

To be held from 3 to 6 June 1986 at München, FR Germany.

*Further information from Münchener Messe-und Austellungsgesellschaft mbH, Messegelände, Postfach 12 10 09, D8000 München 12, FR Germany.*

---

### SAC 86 3rd BNASS: an International Conference on Analytical Chemistry

To be held from 20–26 July 1986 at the University of Bristol, UK.

*Further information from The Secretary, Analytical Division, Royal Society of Chemistry, Burlington House, London W1V 0BN.*

---

*Conference organizers should send full details of their meeting for inclusion in 'Journal of Automatic Chemistry's' calendar at least three months before the event. Information to the Editor, Journal of Automatic Chemistry, Taylor & Francis Ltd, 4 John Street, London WC1N 2ET.*

